# Supplementary material for: The Cell Transformation Assay: A Historical Assessment of Current Knowledge of Applications in an Integrated Approach to Testing and Assessment for Non-Genotoxic Carcinogens
Source: Int J Mol Sci. 2023 Mar 16;24(6):5659. doi: 10.3390/ijms24065659 (PMC10057754; doi:10.3390/ijms24065659)
Supplement: Supplementary file 1 [file ijms-24-05659-s001.zip › Table S1.pdf]

Table S1 – Revised genotoxic data for chemicals able to induce cell transformation in the SHE CTA – Master Table

CAS Registry Number	Chemical name	SHE pH 6.7 [1]	SHE pH 7.0 [1]	Ames	*in vitro* MLA	*in vitro* MN	*in vitro* CA	*in vivo* MN	*in vivo* CA	*in vivo* UDS	*in vivo* transgenic	*in vivo* DNA damage	IARC [2]	CLP	Overall carc. Classification
134-32-7	1-Naphthylamine	-	NA	+	+	NA	+	E	NA	NA	NA	NA	3		N
108-03-2	1-Nitropropane	NA	-	-	+	+	NA	I	-	NA	NA	NA	NA		N
1948-33-0	2 *-tert* -Butyl-1,4-hydroquinone	-	NA	-	NA	NA / +	+	-	-	NA	NA	NA / +	NA		N
93-76-5	2,4,5-Trichlorophenoxyacetic acid	NA	-	-	NA	NA	+	-	+	NA	NA	NA	NA		N
88-06-2	2,4,6-Trichlorophenol	NA	-	-	+	NA	-	NA	NA	NA	NA	NA	2B	C2	Y
95-80-7	2,4-Diaminotoluene	+	+	+	+	NA	+	-	NA	NA / +	NA / +	NA / +	2B	C1B, R2	Y
120-83-2	2,4-Dichlorophenol	NA	-	+	I	NA	E	NA	+	NA	NA	+	NA		N
94-75-7	2,4-Dichlorophenoxyacetic acid	NA	-	-	+	NA	+ / E	-	+	-	NA	NA	2B		N
54150-69-5	2,4-Dimethoxyaniline HCl	-	NA	+	+ / I	NA	+	-	NA	NA	NA	NA / +	NA		N
121-14-2	2,4-Dinitrotoluene	+	-A	+	E	NA	-	-	NA	+	NA	+ / NA	2B	C1B, R2	Y
823-40-5	2,6-Diaminotoluene	-	NA	+	NA / I	+	+	NA / +	NA / -	NA / E	NA / -	NA / -	NA		N
606-20-2	2,6-Dinitrotoluene	+	NA	+	NA	NA	NA	NA	NA	+	NA	+ / NA	2B	C1B, R2	Y
53-96-3	2-Acetylaminofluorene [2-Acetaminofluorene] [2,Fluorenyl acetamide (2-FAA)]	E	NA	+	+	+	+	+ / NA	+ / NA	+ / NA	+ / NA	+ / NA	NA	C1B*	Y
91-59-8	2-Naphthylamine	+	NA	+	I	NA	+	E	NA	+	NA	+	1	C1A	Y
79-46-9	2-Nitropropane	NA	-	+	+	+	E	+ / NA	+ / NA	+	NA	+ / NA	2B	C1B	Y
56-49-5	3-Methylcholanthrene (MCA)	+	+	+	+	+	+ / E	E	NA	E	+	NA	NA	C1B*	Y
504-88-1	3-Nitropropionic acid	-	NA	+	+	-	E	NA	NA	NA	NA	NA	NA		N
28322-02-3	4-Acetylaminofluorene	-	NA	+	E	-	-	NA	-	E	+	NA	NA		N
99-56-9	4-Nitro-*o* -phenylenediamine 4-Nitro-1,2-benzenediamine	-	NA	+	+ / E	NA / -	E / +	E / -	- / NA	NA	NA	NA	3		N

99-55-8	5-Nitro-*o* -toluidine [2-Amino-4-nitrotoluene]	NA	NT	+	NA	NA	+	NA	NA	NA	NA	NA	3	C2	Y
148-24-3	8-Hydroxyquinoline	NA	-	+	I	NA	+	-	NA	- / NA	NA	NA	3	R1B	N
16561-29-8	12-O-Tetradecanoyl phobol 13-acetate (TPA)	+	+	-	-	-	-	NA	NA	NA	NA	NA	NA		NA/Y
3567-69-9	Acid red 14 (IC)	-, +	NA	+	I	NA	-	-	- / NA	NA	NA	NA	3		N
79-06-1	Acrylamide	+	NA	-	+	NA / +	+	+	+	E	+	+	2A	C1B, R2	Y
107-13-1	Acrylonitrile	+	+	+	+	- / NA	+	E	-	-	-	+	2B	C1B	Y
61-82-5	Amitrole [3-Aminotriazole]	NA	I	-	- / I	NA	-	-	-	- / NA	NA	NA	3	R2	Y
7177-48-2	Ampicillin	-, +	NA	-	- / I	NA	-	-	NA / +	NA	NA	NA / -	NA		N
101-05-3	Anilazine	-	NA	-	+	NA / +	-	- / NA	NA	NA	+ / NA	+ / NA	NA		N
120-12-7	Anthracene	-	-	E	+	NA	-	I	NA	NA	NA	NA	3		N
84-65-1	Anthraquinone	-	NA	+	- / NA	+ / NA	+ / NA	+	NA	NA	NA	+ / NA	2B	C1B	Y/N
313-67-7	Aristolochic acid	+	NA	+	+ / NA	+ / NA	+ / NA	+ / E	NA	- / NA	+	+	1		Y
88133-11-3	Bemitradine	+	NA	-	-	NA	-	-	NA	NA	NA	NA	NA		Y
17804-35-2	Benomyl	+,-	NA	-	+	+	NA	+	+	NA	NA	NA	NA	R1B	Y
50-32-8	Benzo(*a*)pyrene	+	+	+	+	+	+	+	+	E	+	E	1	C1B, R1B	Y
119-53-9	Benzoin	NA	-	-	+ / E	-	-	-	-	NA / -	NA	NA / -	NA		N
94-36-0	*Benzoyl peroxide*	NA	+	-	NA	NA	-	NA	NA	NA	NA	NA	3		Y
80-05-7	Bisphenol A	-	NA	-	NE / I	+ / -	E / +	-	+	NA	NA	NA	NA	R1B	N
85-68-7	Butylbenzylphthalate	+	NA	-	-	NA	-	-	+	NA	NA	NA	3	R1B	Y
105-60-2	Caprolactam	-	+	-	-	-	-	-	-	-	NA	NA / -	3		N
120-80-9	Catechol	NA	+	-	+	+	+	+	NA	NA	NA	NA	2B	C1B	Y
302-17-0	Chloral hydrate	+	NA	+	+	+	+	+ / E	- / NA	NA	NA	E / NA	2A		Y
57-74-9	Chlordane Analytical grade	NA	-	-	+	+	-	+	+	NA	NA	+ / -	2B	C2	Y
87-29-6	Cinnamyl anthranilate	-	NA	-	+	NA	-	-	NA	-	NA	NA	3		Y

637-07-0	Clofibrate	+	+	-	NA / -	-	+ / E	NA / -	- / NA	-	NA / I	-	3		Y
477-30-5	Colcemid (IC)	+, +/-	NA	-	NA	+	-	NA	NA	NA	NA	NA	NA		N
5989-27-5	*d* -Limonene	-	I	-	- / I	NA	-	NA	NA	NA	- / I	NA / -	3		Y
69-65-8	*d* -Mannitol	-	NA	-	- / I	NA	-	-	-	NA	NA	NA	NA		N
1163-19-5	Decabromo diphenyloxide	-	NA	-	- / I	NA	-	+	NA / I	NA	NA	NA	3		Y
117-81-7	di (2-Ethylhexyl) phthalate (DEHP)	+	+	-	-	-	-	- / NA	-	-	- / E	-	2B	R1B	Y
439-14-5	Diazepam	-, +/-	NA	-	NA	+	-	+	I	NA	NA	NA	3		N
50-29-3	Dichlorodiphenyl trichloroethane (DDT)	NA	-	-	-	+	I	-	+	-	NA	+	2A	C2	Y
111-42-2	Diethanolamine	+	NA	-	- / I	NA	-	-	NA	NA	NA	NA / -	2B		Y
56-53-1	Diethylstilbestrol (DES)	E	E	-	+ / I	+	+	E	+	NA	NA	NA / -	1	C1B*, R1B*	Y
150-38-9	EDTA, trisodium salt trihydrate	-	NA	-	- / I	NA	-	NA	NA	NA	NA	NA / -	NA		N
106-89-8	Epichlorhydrin	+	NA	+	+	NA	+	-	E	+	NA	NA	2A	C1B	Y
64-17-5	Ethyl alcohol [Ethanol]	-	NA	-	-	NA	-	E	-	NA	NA	+	1		Y
100-41-4	Ethyl benzene	+	NA	-	+	+	-	-	NA	-	NA	NA	2B		Y
60-00-4	Ethylenediaminetetracetic acid	-	NA	-	+	NA	-	NA	NA	NA	NA	NA	NA		N
97-53-0	Eugenol	-	NA	-	+	NA / +	+	E / -	NA	NA / -	NA / -	NA	3		N
67774-32-7	Firemaster FF1	NA	NA	NA	-	NA	NA	-	-	-	NA	-	NA		Y
50-00-0	Formaldehyde	+	NA	+	+	+ / NA	+	E	-	NA	NA	NA	1	C1B	Y
98-00-0	Furfuryl alcohol	-	NA	-	NA	NA	E	-	- / E	NA	NA	NA	2B	C2	Y
446-72-0	Genistein	-	NA	-	+	+	+	-	-	NA	NA	NA	NA		Y
126-07-8	Griseofulvin	-, +	NA	-	+	+	+	-	+	NA	NA	NA	2B	C2*, R1B*	Y
33229-34-4	HC Blue 2	-	NA	+	I / +	+	-	-	- / NA	-	NA	NA	3		N
680-31-9	Hexamethyl phosphoramide	NA	+	E / +	E	+	E	+	+	NA	NA	NA	2B	C1B	Y

123-31-9	Hydroquinone	NA	+	-	+	+	+	+	+	NA	NA	NA	3	C2	Y
78-84-2	Isobutyraldehyde	-	NA	-	+	NA / -	+	-	+	NA	NA	NA / -	NA		N
50-81-7	l-Ascorbic acid	-	NA	E / +	E	+	-	+	+	NA	NA	-	NA		N
5874-97-5	Metaproterenol hemisulfate	-	NA	-	-	NA	-	-	NA	NA	NA	NA	NA		Y
135-23-9	Methapyrilene HCl	+	NA	+	I	NA	+	NA	NA	NA	NA	NA	NA		Y
72-43-5	Methoxychlor	+	NA	-	+	NA	-	NA	NA	NA	NA	NA	3		N/Y
93-15-2	Methyl eugenol	+	NA	-	NA / -	NA / -	I / -	-	NA	NA	NA / +	NA / +	2B	C2*	Y
598-55-0	Methylcarbamate (Methyl urethane)	-	NA	-	- / I	NA	-	-	NA	NA	NA	NA / -	3	C2*	Y
21340-68-1	*Methylclofenapate*	NA	+	-	NA	NA	NA	NA	NA	-	NA	NA	NA		Y
105-55-5	*N* ,*N’* -Diethylthiourea	+	NA	-	+	NA	-	NA	NA	NA	NA	NA	3		Y
70-25-7	*N* -Nitroso-*N* -methylnitroguanidine [N- Methyl-N’-nitro-N-nitrosoguanidinen (MNNG)]	+	NA	+	+	+	+	+	+	+	+	+	2A	C1B	Y
141-32-2	n-Butyl acrylate	NA	-	-	+	-	+	NA	+	NA	NA	NA	3		N
139-13-9	Nitrilotriacetic Acid	-, +	NA	-	- / E	+	- / E	-	NA	NA	NA	+	2B	C2*	Y
90-04-0	*o* -Anisidine	+	I	- / +	+	NA	+	-	NA	-	+	+	2A	C1B	Y
134-29-2	*o* -Anisidine HCl	NA	NA	+	NA / +	NA	NA / +	-	NA	NA / -	NA / +	NA / +	2A	C2*	Y
95-53-4	*o-* Toluidine	+	+	+	+ / I	+ / NA	+ / E	- / +	+ / -	NA	NA	+	1	C1B	Y
636-21-5	*o* -Toluidine HCl	NA	NA	E / +	NA / I	I / NA	I / E	I / +	I / -	NA	NA	NA / +	1	C1B*	Y
434-07-1	Oxymetholone	+	NA	-	-	NA / +	-	-	- / NA	NA	NA	NA	NA	C2*, R2*	Y
6493-05-6	Pentoxifylline	-	NA	-	+	+	+	NA	NA	NA	NA	NA	NA		N
62-44-2	Phenacetin	-	NA	+	I	-	+	+	- / NA	NA	NA	+	1	C1B*	Y

50-06-6	Phenobarbital	+	+	+	I	-	+	E	-	-	E	NA	2B	C2*	Y
57-30-7	Phenobarbital sodium	NA	NA	-	NA	NA	NA	-	NA	NA	-	+	NA	C2*	Y
108-95-2	Phenol	NA	-	-	E / +	+	+ / NA	E / +	+ / -	NA	NA	+	3		N
77-09-8	Phenolphthalein	+	NA	-	NA	-	+	+	NA	NA	NA	-	2B	C1B, R2	Y
85-44-9	Phthalic anhydride	E	-	-	+ / I	NA	-	NA / I	NA	NA	NA	NA	NA		N
624-18-0	p-Phenylenediamine 2HCl 4-Aminoaniline dihydrochloride	-	NA	+	+	+	+	-	NA	NA / -	NA	-	NA		N
125-33-7	Primidone	-	NA	+	- / NA	+ / NA	-	-	NA	NA	NA	NA	2B	C2*	Y
75-56-9	Propylene oxide	+	NA	+	+	+ / NA	+	+ / E	+	NA	NA	NA	2B	C1B	Y
110-86-1	Pyridine	-	NA	-	- / I	NA	-	-	- / NA	-	NA	- / NA	2B		Y
50-55-5	Reserpine	+	NA	-	- / I	NA / -	-	- / +	+	-	NA	NA	3		Y
108-46-3	Resorcinol	-	-	-	+	NA / +	+	+ / -	NA	NA	NA	NA	3		N
83-79-4	Rotenone	+	NA	-	+	+	+	+ / -	NA	NA	NA	NA	NA		N
1300-72-7	Sodium xylenesulfonate	-	NA	-	E	NA	-	NA	NA	NA	NA	NA	NA		N
127-69-5	Sulfisoxazole	-	NA	-	+ / I	NA / -	-	-	NA	NA	NA	NA	3		N
97-77-8	Tetraethylthiuramdisulfide Disulfiram	+	NA	-	+	NA	+	- / I	- / NA	NA	NA	NA	3		N
109-99-9	Tetrahydrofuran	-	NA	-	NA / I	NA / -	-	E	-	-	NA	NA	2B	C2	Y
148-79-8	ThiabeNAazole	-	NA	+	+	E	+	+	NA	NA	NA	+	NA		N
76-87-9	Triphenyltin hydroxide	-	NA	-	+ / E	NA / +	-	NA / I	NA	NA	NA	NA	NA	C2, R2	N
78-42-2	Tris(2-ethylhexyl)phosphate (TEHP)	-	NA	-	- / I	NA	-	-	-	NA	NA	NA	NA		Y
51-79-6	Urethane [Ethyl carbamate]	+	NA	E / +	-	-	E	+	+	+ / NA	+	E / NA	2A	C1B	Y
50892-23-4	Wyeth-14,643	+	NA	-	NA	+	NA / +	-	NA	NA / -	NA / +	NA / +	NA	C1B*	Y

The genotoxicity data collected in the initial evaluation by the OECD CTA expert group, were updated considering the more recently published and curated EURL ECVAM Genotoxicity and Carcinogenicity Consolidated Database of Ames Negative Chemicals [127] (chemicals highlighted in green). The data from the non-highlighted chemicals

could not be revised since they were not present in the aforementioned database. The red highlights represent the cases in which the original data differed from the ones in the database.

+: positive; -: negative; NA: not available; E: equivocal; I: inconclusive (inadequate evidence); #: dose at pH 7 were very low compared to the study at pH 6.7; Y: carcinogen; N: non-carcinogen; *: non-harmonised classification

[1] OECD (2007). Detailed Review Paper on Cell transformation assays for detection of chemical carcinogens, OECD Environment, Health and Safety Publications, Series on Testing and Assessment, No. 31. OECD, Paris [37]

[2] Reviewed according to 2019 update to the IARC Monographs Preamble [128]
