# Supplementary material for: The Cell Transformation Assay: A Historical Assessment of Current Knowledge of Applications in an Integrated Approach to Testing and Assessment for Non-Genotoxic Carcinogens
Source: Int J Mol Sci. 2023 Mar 16;24(6):5659. doi: 10.3390/ijms24065659 (PMC10057754; doi:10.3390/ijms24065659)
Supplement: Supplementary file 1 [file ijms-24-05659-s001.zip › Table S2.pdf]

Table S2 – Revised genotoxic data for chemicals able to induce cell transformation in the SHE CTA , final list of chemicals

CAS Registry Number	Chemical name	SHE pH 6.7	SHE pH 7.0	Ames	*in vitro* MLA	*in vitro* MN	*in vitro CA*	*in vivo* MN	*in vivo* CA	*in vivo* UDS	*in vivo* transgenic	*in vivo* DNA damage	IARC[1]	CLP (CMR)	NTP (RoC)[2]	Overall carc. Classification	Use
88133-11-3	**Bemitradine**	+	NA	-	-	NA	-	-	NA	NA	NA	NA	NA		+ (NL)	Y/Y	Diuretic antihypertensive drug
94-36-0	***Benzoyl peroxide***	NA	+	-	NA	NA	-	NA	NA	NA	NA	NA	3		-	Y/Y	Drug product
85-68-7	**Butylbenzylphthalate**	+	NA	-	-	NA	-	-	+	NA	NA	NA	3	R2 / R1B	I/ (NL)	Y/Y	Plasticizer
637-07-0	**Clofibrate**	+	+	-	-	-	E	-	-	-	I	-	3		-	Y/Y	Antilipidemic aNA anticholesteremic drug
117-81-7	**di (2-Ethylhexyl) phthalate (DEHP)**	+	+	-	-	-	-	-	-	-	E	-	2B	R1B	NA	Y/Y	Plasticizer
111-42-2	**Diethanolamine**	+	NA	-	I	NA	-	-	NA	NA	NA	-	2B		Reasonably anticipated to be carcinogenic (RoC)	Y/Y	Emulsifier
105-55-5	***N ,N′*** **-Diethylthiourea**	+	NA	-	+	NA	-	NA	NA	NA	NA	NA	3		+ in mouse both sexes, but not in rats (NL)	Y/Y	Rubber chemical
100-41-4	**Ethyl benzene**	+	NA	-	+	+	-	-	NA	-	NA	NA	2B		+ in rats both sexes, but not in mouse (NL)	Y/Y	Intermediate
93-15-2	**Methyl eugenol**	+	NA	-	-	-	-	-	NA	NA	+	+	2B	M2*, C2*	LE (NL)	Y/Y	Flavoring agent
21340-68-1	**Methylclofenapate**	NA	+	-	NA	NA	NA	NA	NA	-	NA	NA	NA		-	Y/Y	Antilipidemic drug
434-07-1	**Oxymetholone**	+	NA	-	-	+	-	-	-	NA	NA	NA	NA	C2*, R2*	Reasonably anticipated to be carcinogenic (RoC)	Y/Y	Anabolic steroid

50-55-5	**Reserpine**	**+**	**NA**	**-**	**I**	**-**	**-**	**+**	**+**	**-**	**NA**	**NA**	**3**		**NA**	**Y/Y**	Antihypertensive drug
50892-23-4	**Wyeth-14,643**	**+**	**NA**	**-**	**NA**	**+**	**+**	**-**	**NA**	**-**	**+**	**+**	**NA**	**C1B ***	**Reasonably anticipated to be carcinogenic (RoC)**	**Y/Y**	Drug product
16561-29-8	**12-O-Tetradecanoyl phobol 13-acetate (TPA)**	**+**	**+**	**-**	**-**	**-**	**-**	**NA**	**NA**	**NA**	**NA**	**NA**	**NA**		**Reasonably anticipated to be carcinogenic (RoC)**	**NA/Y**	Phorbol ester
72-43-5	**Methoxychlor**	**+**	**NA**	**-**	**+**	**NA**	**-**	**NA**	**NA**	**NA**	**NA**	**NA**	**3**		**Positive (NL)**	**N/Y**	Pesticide
105-60-2	**Caprolactam**	**-**	**+**	**-**	**-**	**-**	**-**	**-**	**-**	**-**	**NA**	**-**	**3**		**Negative**	**N/N**	Used in nylon manufacture

+: positive; -: negative; NA: not available; E: equivocal; I: inconclusive (inadequate evidence); *: non-harmonised classification; LE = Limited Evidence;

[1] Reviewed according to 2019 update to the IARC Monographs Preamble [128]

[2] Updated according to 15th Report on Carcinogens [129]
